# Factors Associated With Patient Failure To Attend Dental Appointments: A Retrospective Analysis

**DOI:** 10.7759/cureus.67061

**Published:** 2024-08-17

**Authors:** Khalid Alkhurayji, Abdullah Alharbi, Ibrahim Walbi, Ahmed AlShpep, Yousef Almuhawwis, Abdullah Alalwan, Alya Alnoaimi, Abdulrahman Redwan, Ghadeer Alshehri, Naif Almalki

**Affiliations:** 1 Dental Center, Prince Sultan Military Medical City, Riyadh, SAU

**Keywords:** dental clinic, dental services, non-attendance, dental appointment, hospital dentistry

## Abstract

Nonattendance at scheduled dental appointments has a significant impact on healthcare systems worldwide. This study examines the factors associated with missed appointments at the Dental Center in the Department of Oral and Dental Health in Riyadh, Saudi Arabia, through a retrospective secondary data analysis. Existing medical records from January 1, 2024, to May 1, 2024 were analyzed to identify patterns or factors contributing to nonattendance. Data were collected using a standardized sheet and analyzed with statistical methods, including correlation analysis, ANOVA, and chi-square tests, to determine significant associations and factors affecting nonattendance. The results indicated that the majority of nonattendees were single (56.2%), with a higher proportion of females (60.7%) compared to males (39.3%). Only 3.8% of those who missed their appointments were over 55 years old. Tuesdays had the highest incidence of nonattendance (331 cases). No significant association was found between age groups and the time (F = 0.224, p = 0.925) or date (F = 0.840, p = 0.500) of appointments. Patients were less likely to attend morning appointments compared to evening ones. The high rate of missed appointments reduces the effectiveness and efficiency of the Dental Center’s resources. The identified patterns and factors can guide managers and policymakers in developing strategies to reduce missed appointments and improve overall appointment adherence.

## Introduction

Nonattendance at scheduled dental appointments significantly impacts healthcare systems worldwide [1-3]. Globally, there is a persistent demand for cost reduction, coupled with pressures to enhance the efficiency of dental services [4-6]. Understanding the factors associated with nonattendance is crucial for developing strategies to improve appointment adherence and optimize dental care [7,8]. Furthermore, the increasing rate of nonattendance exacerbates the challenge of fully utilizing healthcare services [9,10].

A previous systematic review identified young age, insurance coverage, and distance from the clinic as key factors contributing to nonattendance at dental appointments. Additionally, a history of previous nonattendance was significantly associated with future nonattendance [11,12]. A study conducted in Saudi Arabia emphasized the importance of retrospective data analysis in predicting nonattendance rates [13].

Understanding the factors that lead to patient nonattendance is crucial for developing strategies to improve appointment adherence and enhance the efficiency of patient care delivery. This study aims to determine the factors affecting patient nonattendance at the Dental Center in the Department of Oral and Dental Health.

## Materials and methods

Study design

This study employed a retrospective secondary data analysis design to examine the association between various factors and nonattendance among patients receiving dental services at the Dental Center, Department of Oral and Dental Health in Riyadh, Saudi Arabia. The research involved collecting and analyzing existing medical records to identify patterns or factors contributing to patient nonattendance.

Study setting and population

The target population for this study comprised patients accessing dental services at the Dental Center, Department of Oral and Dental Health in Riyadh City. It included all patients with scheduled appointments at the Dental Center during the study period from January 1, 2024, to May 1, 2024. Inclusion criteria were patients of all ages and genders with appointments documented in the center's records, including those with a history of nonattendance. Exclusion criteria were patients without documented appointment records at the Dental Center during the study time frame and those with incomplete or missing information in their appointment records.

Data collection

The data was collected using a standardized data sheet, which facilitated the extraction of relevant information from patients’ medical records. This approach ensured accuracy and consistency in capturing variables related to patient nonattendance. The standardized data sheet was designed to record variables such as appointment dates and times, patient demographics, previous attendance history, and any pertinent clinical information that might have influenced appointment adherence. The process involved systematically reviewing appointment records to identify patterns of nonattendance. Data regarding nonattendance was then recorded and coded for subsequent analysis to assess associations and trends.

Data analysis

The independent variables in the analysis included demographics (e.g., age, gender, and health status), previous appointment attendance history, appointment times, and the type of dental service scheduled.

Data analysis was conducted using IBM SPSS Statistics for Windows, Version 25.0 (Released 2017; IBM Corp., Armonk, NY, USA). The focus was on assessing the associations between these variables and patient nonattendance. Statistical methods such as correlation analysis, ANOVA, and chi-square tests were employed to identify significant associations and factors influencing nonattendance. Descriptive statistics summarized the characteristics of the study population, while inferential statistics determined the strength and direction of relationships between the independent variables and patient nonattendance.

Ethical considerations

Ethical considerations are crucial in research involving patient data. This study ensured ethical integrity by taking several precautions to protect patient confidentiality and privacy. Written consent was not required, as the data was de-identified and used solely for research purposes. All procedures followed the principles of the Declaration of Helsinki and received approval from the Institutional Review Board of the Prince Sultan Military Medical City and Dental Center. Data protection regulations were strictly adhered to, and the research team was trained in handling sensitive information and maintaining data security. Results were reported in aggregate form to maintain patient anonymity and uphold ethical research practices.

## Results

The results revealed that the majority of patients who did not attend their appointments were single (56.2%), with females accounting for 60.7% of this group. Among the nonattending patients, healthy individuals (59.4%) were more common than those with medical issues. The largest age group of nonattendees were those under 25 years old (36.7%), followed by patients aged 26 to 35 (30.6%). Only 3.8% of nonattendees were over 55 years old (Table 1).

**Table 1:** Characteristics of patients who failed to attend appointments

Characteristic	N	%
Marital status		
Married	591	43.3
Single	767	56.2
Divorced	2	0.1
Widow	4	0.3
Total	1,364	100
Gender		
Male	536	39.3
Female	828	60.7
Total	1,364	100
Health status		
Fit	810	59.4
Unfit	554	40.6
Total	1,364	100
Age (years)		
≤25	500	36.7
26-35	417	30.6
36-45	271	19.9
46-55	124	9.1
≥56	52	3.8
Total	1,364	100

**Figure 1:**
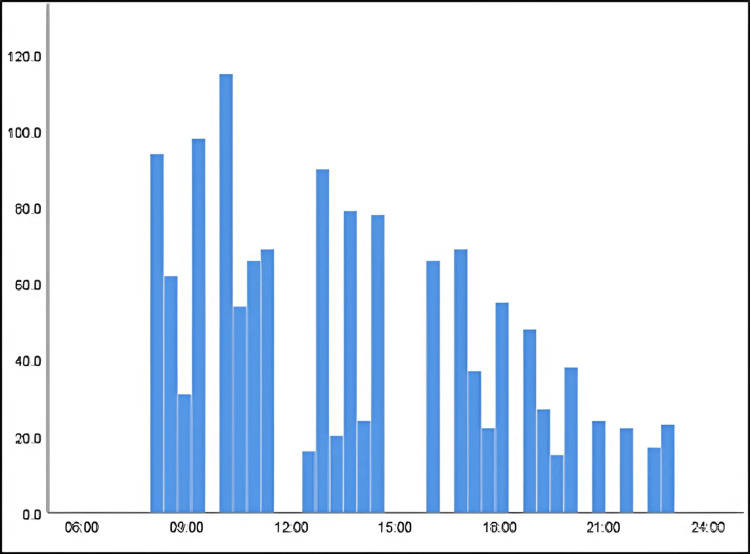
Periods of nonattendance

Figure 1 illustrates the times of day during which patients failed to attend appointments. Nonattendance was most common for morning appointments between 8:00 AM and 3:00 PM, with fewer instances of nonattendance occurring from 4:00 PM to 8:00 PM.

Table 2 illustrates the characteristics of nonattending patients by day of the week. Tuesdays exhibited the highest incidence of nonattendance (331 cases), while nonattendance was similar on Mondays (274 cases), Wednesdays (273 cases), and Thursdays (251 cases). Sunday had the lowest rate of nonattendance (234 cases). Additionally, married individuals were less likely to miss appointments on Thursdays compared to other days.

**Table 2:** Descriptive statistics of patients who failed to attend appointments

Variable	Sunday	Monday	Tuesday	Wednesday	Thursday	p-value
	N (%)	N (%)	N (%)	N (%)	N (%)	
Marital status						
Married	100 (42.6)	116 (42.3)	140 (42.3)	116 (42.5)	119 (47.4)	
Single	134 (57.0)	156 (56.9)	190 (57.4)	156 (57.1)	131 (52.2)	
Divorced	0 (0.0)	1 (0.4)	1 (0.3)	0 (0.0)	0 (0.0)	0.919
Widow	1 (0.4)	1 (0.4)	0 (0.0)	1 (0.4)	1 (0.4)	
Total	234 (100)	274 (100)	331 (100)	273 (100)	251 (100)	
Gender						
Male	98 (41.7)	104 (38.0)	127 (38.4)	106 (38.8)	101 (40.2)	
Female	137 (58.3)	170 (62.0)	204 (61.6)	167 (61.2)	150 (59.8)	0.908
Total	234 (100)	274 (100)	331 (100)	273 (100)	251 (100)	
Health status						
Fit	136 (57.9)	161 (58.8)	201 (60.7)	163 (59.7)	149 (59.4)	
Unfit	99 (42.1)	113 (41.2)	130 (39.3)	110 (40.3)	102 (40.6)	0.971
Total	234 (100)	274 (100)	331 (100)	273 (100)	251 (100)	
Age						
≤25	78 (33.2)	99 (36.1)	123 (37.2)	107 (39.2)	93 (37.1)	
26-35	80 (34.0)	92 (33.6)	97 (29.3)	79 (28.9)	69 (27.5)	
36-45	45 (19.1)	52 (19.0)	68 (20.5)	50 (18.3)	56 (22.3)	0.954
46-55	24 (10.2)	21 (7.7)	32 (9.7)	26 (9.5)	21 (8.4)	
≥56	8 (3.4)	10 (3.6)	11 (3.3)	11 (4.0)	12 (4.8)	
Total	234 (100)	274 (100)	331 (100)	273 (100)	251 (100)	
Visit type						
New	202 (86.0)	228 (83.2)	257 (77.6)	222 (81.3)	203 (80.9)	
Follow-up	33 (14.0)	46 (16.8)	74 (22.4)	51 (18.7)	48 (19.1)	0.137
Total	234 (100)	274 (100)	331 (100)	273 (100)	251 (100)	

Gender analysis showed no significant variation in nonattendance across the days of the week, with male nonattendance rates ranging from 38% to 41.7% and female rates from 58.3% to 62%. Regarding appointment type, new patients were more likely to miss appointments on Sunday (86%) compared to follow-up patients (14%). Monday also had a high rate of nonattendance among new patients (83.2%). The association test indicated no significant relationship between patient characteristics and the days of the week.

**Figure 2:**
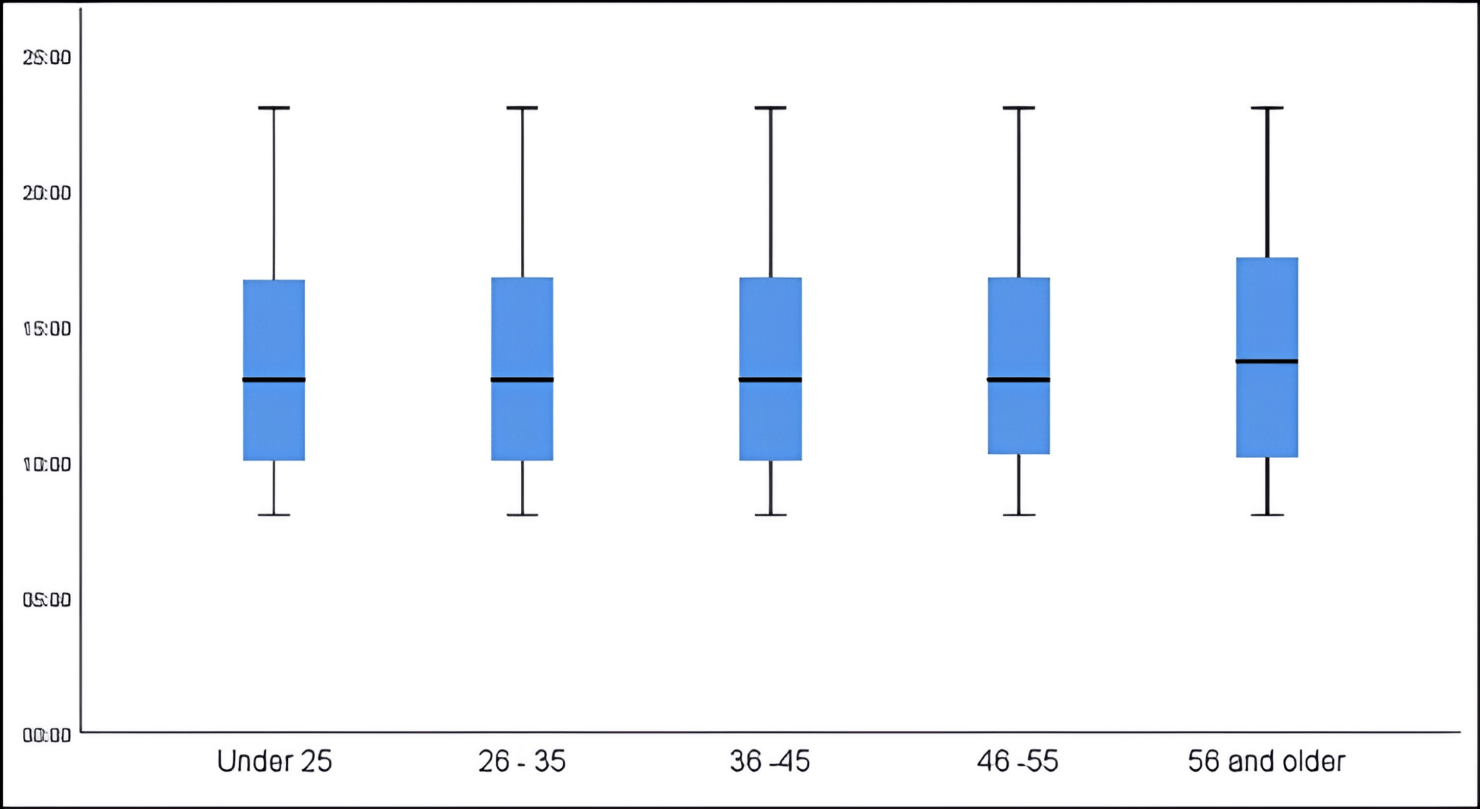
Time of nonattended appointments by patient age group

Figure 2 illustrates the times of day when appointments were missed, categorized by patient age group. There was no significant association between age group and the time of appointment (F = 0.224, p-value = 0.925).

**Figure 3:**
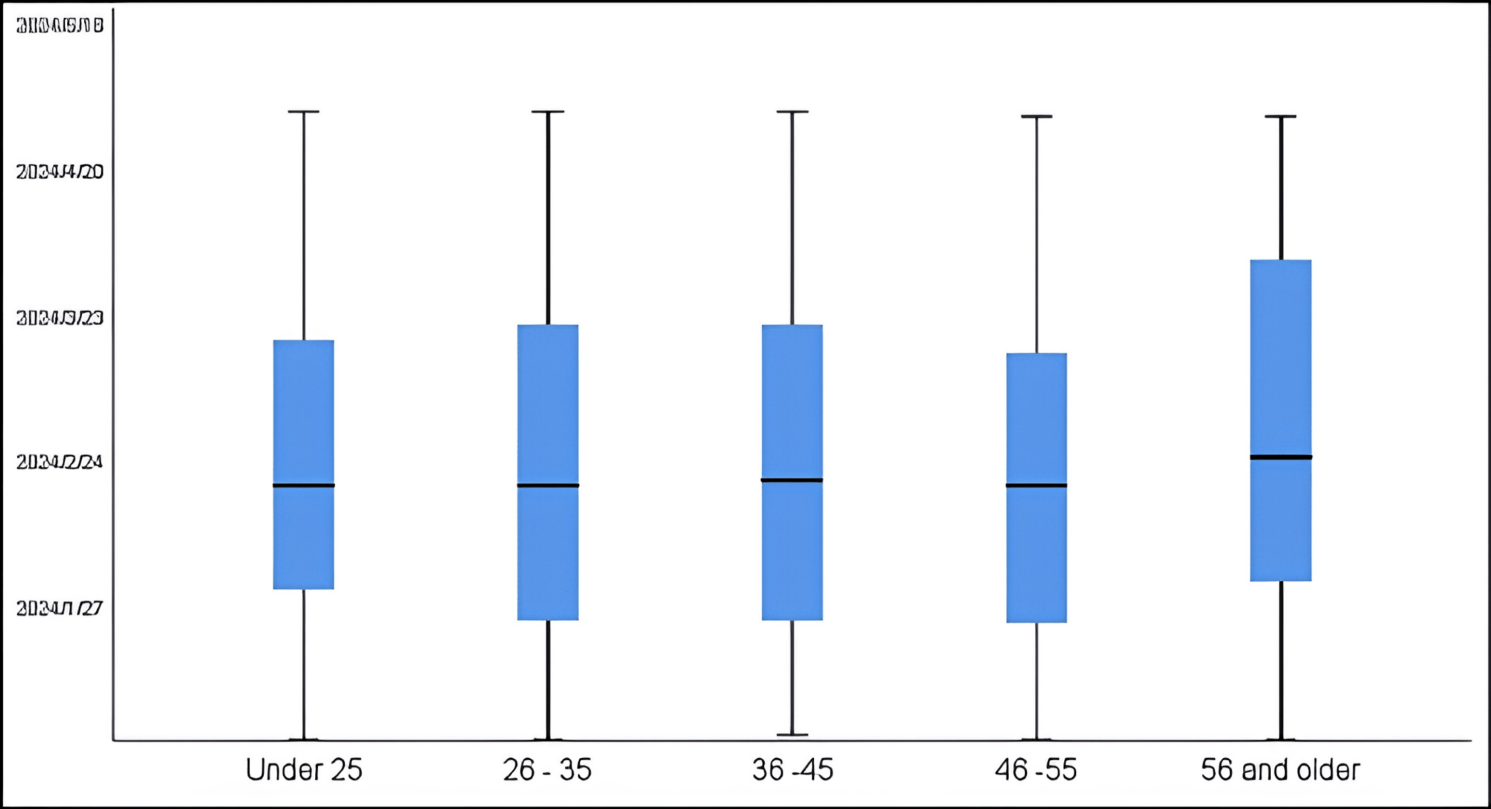
Date of nonattended appointments by patient age group

Figure 3 illustrates the dates of nonattended appointments by patient age group. There was no significant association between age group and date of appointment (F = 0.840, p-value = 0.500). A positive relationship was observed between age and the date of appointment (r = 0.029), while appointment time showed a negative correlation with age (r = -0.011).

## Discussion

The study found that neither the time nor the date of the visit was significantly associated with the patient’s age, and no relationship was observed between the days of the week and patient characteristics. The analysis revealed that single patients aged 35 and under were most likely to miss dental clinic appointments. Additionally, patients were less likely to attend morning appointments compared to those scheduled later in the day.

Alkhuwaiter et al. suggest that fear and anxiety might contribute to missed first appointments [14]. This study supports this, noting a pattern of nonattendance at initial visits. Furthermore, previous research from provincial hospitals indicated that patients often missed appointments due to difficulties taking time off work, which aligns with our finding that morning appointments had higher nonattendance rates [15].

Younger patients are more likely to miss appointments, as highlighted in studies from various regions. However, this study did not find a correlation between age and appointment time [16]. Factors such as the date of the appointment may also influence nonattendance rates. Bhatia et al. identified multiple factors, including appointment timing, as contributing to missed dental visits [17].

To our knowledge, this study represents the first retrospective secondary analysis of dental appointment nonattendance conducted among Riyadh City dental centers, addressing gaps in both demographic and methodological knowledge. Despite its contributions, the study has limitations. Expanding the analysis to include more variables and extending the study period to a full year could provide a more comprehensive understanding of nonattendance patterns.

To reduce appointment no-shows, clinics could consider offering evening appointments, which may better accommodate patients’ work schedules. Additionally, providing walk-in slots could help decrease missed appointments.

Future research should focus on further retrospective analyses to explore additional variables and improve the generalizability of the findings. Continued efforts in this area could enhance the efficiency of dental care services and resource utilization.

## Conclusions

This study revealed a high rate of nonattendance at dental appointments, affecting the effectiveness and efficiency of the Dental Center’s resources. The identified patterns and variables offer valuable insights for managers and policymakers to design interventions aimed at reducing missed appointments. By highlighting the characteristics of patients and the timing of appointments, the study provides clear recommendations for enhancing the efficiency and effectiveness of the appointment system.
